# (La_0.65_Sr_0.3_)_0.95_FeO_3−*δ*_ perovskite with high oxygen vacancy as efficient bifunctional electrocatalysts for Zn–air batteries†

**DOI:** 10.1039/d1ra07920d

**Published:** 2021-12-06

**Authors:** Liyang Luo, Zhongyi Liu, Zhiyuan Wang

**Affiliations:** College of Chemistry, Zhengzhou University Zhengzhou 450001 China; Henan Institute of Advanced Technology, Zhengzhou University Zhengzhou 450001 China wangzhiy@zzu.edu.cn; Institute of Physical Chemistry, RWTH Aachen University 52074 Aachen Germany

## Abstract

Developing low-cost, highly efficient electrocatalysts for the oxygen reduction reaction (ORR) and oxygen evolution reaction (OER) is desirable for rechargeable metal–air batteries. Herein, a series of perovskite structured (La_0.65_Sr_0.3_)_0.95_FeO_3−*δ*_ catalysts with A-site deficiency were synthesized through a scalable solid state synthesis method at different calcination temperatures. The electrocatalytic activities of these catalysts were investigated by thin-film RDE technique. The catalyst calcined at 1000 °C exhibits an outstanding bi-functional activity towards the ORR and OER in alkaline electrolyte, and it also exhibits an outstanding performance in primary and rechargeable Zn–air batteries, which is comparable with the commercial noble metals Pt/C and RuO_2_.

## Abstract 2

Developing low-cost, highly efficient electrocatalysts for the oxygen reduction reaction (ORR) and oxygen evolution reaction (OER) is desirable for rechargeable metal–air batteries.

## Graphical abstract

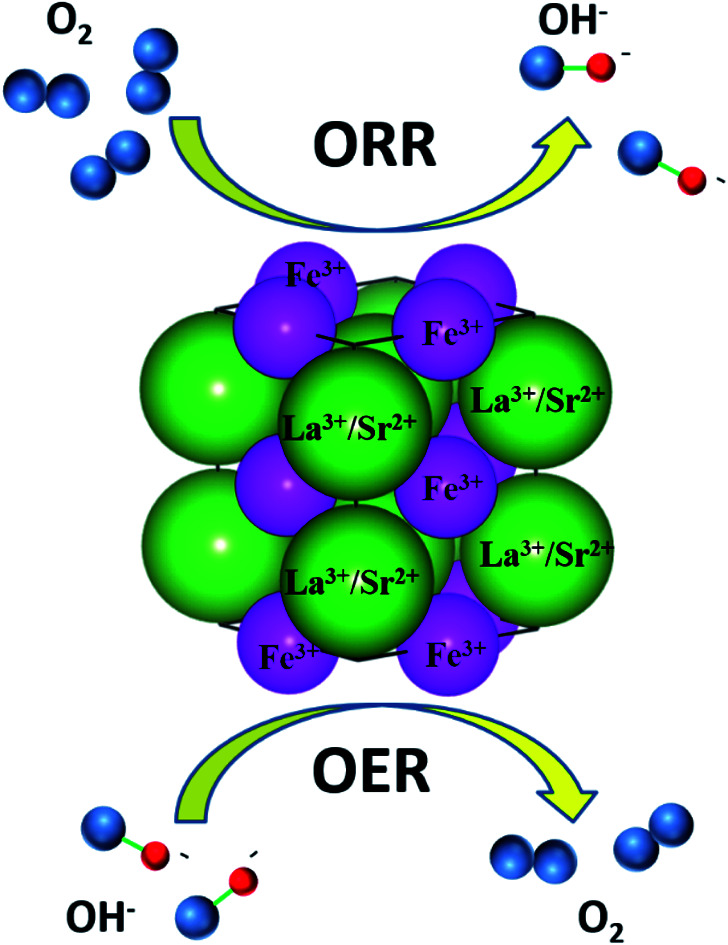

Metal–air batteries have attracted great attention because of their high energy and power densities.^1–3^ Of all the metal–air batteries, the Zn–air battery is particularly attractive and considered to be an alternative battery owing to its long cycle life, low cost and the global abundance of zinc.^4–7^ Furthermore, the specific energy of the Zn–air battery is 1084 W h kg^−1^, which is quite attractive for applications. However, to achieve a Zn–air battery with high performance and long cycle life, an efficient and highly stable bifunctional air electrode is essential, because the sluggish kinetics of the oxygen reduction reaction (ORR) and the oxygen evolution reaction (OER) during discharge and charge have limited the practical application of Zn–air batteries.^8,9^ To solve this problem, a variety of highly active ORR and OER electrocatalysts have been investigated, including noble metals,^10,11^ carbon materials,^12–14^ transition metal selenide,^15^ and transition metal oxides.^16–18^ Noble metals exhibit high performance, but their low abundance and high cost prohibit their particle use. The carbon materials possess larger surface areas and high conductivities, but poor durability.

Transition metal oxides, especially the perovskite structured oxides have been intensively investigated as bifunctional electrocatalysts in Zn–air batteries, owing to their low-cost, abundant varieties, structural stability and great potentials.^19–21^ As far as we know, in perovskite catalysts, the A-site deficiency and substitute A-site metal with low valence metals can manipulate the B-site element valence, and which has a critical influence on the catalytic behaviours of the materials. Herein, a series of perovskite structured catalysts are designed with same A-site stoichiometry and same content of doping metals, but calcined at different temperatures. The relationship between the preparation temperature and the oxidation state of B-site metal is investigated; and it obviously affect their catalytic activities towards ORR and OER in alkaline medium.^22–24^ The selected catalyst can be used as bifunctional electrocatalyst and is comparable with commercial noble metals.

The LSF catalysts prepared at different temperature are characterized by X-ray diffraction (XRD), and the results are shown in Fig. 1(a). Compared with the standard PDF pattern (PDF: 01-089-1269), all the characteristic peaks can be well indexed as a perovskite phase with an orthorhombic structure. But the sample prepared at 900 °C exhibits two small peaks around 30° correspond to the secondary phase of La_2_O_3_. To avoid the effect of La_2_O_3_ impurity phase, the sample calcined at 900 °C is not selected for further investigated. To confirm the chemical compositions of the LSF0.95 catalysts, inductively coupled plasma with optical emission spectroscopy (ICP-OES) is used and the results are shown in Table S1.† As shown in Table S1,† the chemical compositions of the LSF0.95 catalysts agree well with the designed compositions. SEM images in Fig. 1(d) show the microstructure and the surface morphology of LSF perovskite catalysts calcined at different temperatures. The low magnification SEM images reveal that the LSF particles are homogeneously distributed for all LSF catalysts. The high magnification SEM images and TEM image (Fig. 1(f)) clearly exhibit agglomeration of the particles with irregularly shapes, and with increasing of the calcination temperature from 1000 °C to 1200 °C, a gradual increase of the particle size can be observed, and the surface of the agglomerated particles becomes smoother. The particle size for the LSF0.95-1000 °C, LSF0.95-1100 °C and LSF0.95-1200 °C is ∼0.8–2.5 μm, ∼0.8–3 μm, and ∼1–3.4 μm, respectively. These values are close to the results of particle size distribution (PSD) shown in Fig. S1.† Energy dispersive X-ray (EDX) mapping in Fig. 1(e) represent that La, Sr, Fe and O are homogeneous distributed in the LSF0.95 catalysts. The high-resolution transmission electron microscopy (HRTEM) image in Fig. 1(g) and corresponding SAED pattern in Fig. 1(h) clearly exhibit the 020 crystal phase of LSF0.95-1000 °C. The specific surface areas of LSF0.95 catalysts are determined from the nitrogen adsorption/desorption isotherms. As shown in Fig. S2,† the adsorption and desorption isotherms are almost coincided with each other, indicating no mesopores in these LSF catalysts. The specific surface area of LSF catalysts are calculated by BET model (Table S2†), the values are 1.441 m^2^ g^−1^ (LSF0.95-1000 °C), 1.070 m^2^ g^−1^ (LSF0.95-1100 °C) and 0.816 m^2^ g^−1^ (LSF0.95-1200 °C), respectively. These values represent that the higher calcination temperature leads to the smaller specific surface area. Because the particles are easier to agglomerate at higher calcination temperature, resulting in the bigger particle size, which has been discussed in SEM and PSD. The surface element compositions of the LSF catalysts are analyzed by X-ray photoelectron spectroscopy (XPS). The survey XPS spectrum in Fig. S3(a)† confirms the existence of La, Sr, Fe and O in the LSF0.95 catalysts. In Fig. S3(b),† the peaks centered at 710.3 eV and 723.5 eV are corresponding to the binding energy of Fe 2p_3/2_ and Fe 2p_1/2_, respectively;^25,26^ and the weak Fe-2p satellite peak indicates the multiple oxidation states of Fe. High-resolution XPS spectra of Fe 2p_3/2_ and O 1s are analyzed in Fig. 1 and the chemical compositions are summarized in Table S4.† Fe 2p_3/2_ in Fig. 1(b) reveal that the oxidation state of Fe in LSF catalysts are mixed by Fe^3+^ (709–711 eV) and Fe^4+^ (711–713.6 eV), and the content of Fe^4+^ increases as the temperature increasing from 1000 °C to 1200 °C. The peaks at 528.5 eV, 529.9 eV and 531.5 eV (Fig. 1(c)) in O 1s are corresponding to the crystal lattice oxygen species, adsorbed oxygen, and adsorbed water, respectively.^27–30^

**Fig. 1:**
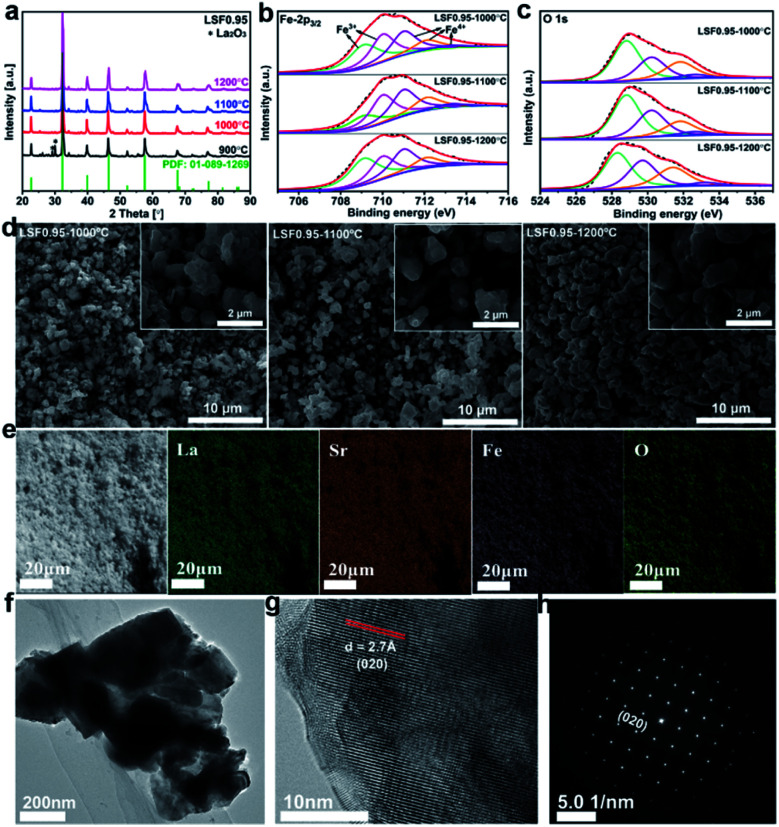
XRD patterns (a) and XPS spectra of Fe 2p species (b) and O 1s species (c) of the LSF0.95-1000 °C, LSF0.95-1100 °C and LSF0.95-1200 °C. SEM images (d) and EDX maps (e) of the LSF0.95 catalysts. (f) TEM image, (g) TRTEM image and (h) SAED pattern of LSF0.95-1000 °C.

Electrocatalytic activities of the LSF0.95 catalysts towards ORR are investigated by thin-film RDE technique. Fig. S4† exhibits the comparison of cyclic voltammetry (CV) curves obtained in O_2_ and N_2_ saturated KOH. Two obvious cathodic peaks centered at 0.34 V and −0.02 V (*vs.* RHE) only can be observed in CV curve tested in O_2_ saturated KOH, indicating the electrocatalytic activity of LSF catalyst towards ORR in KOH. LSV curves of the LSF catalysts with different preparation temperatures measured at 1600 rpm are compared in Fig. 2(a), and all the LSV curves possess two ORR limiting current plateaus, which are in accordance with the two reduction peaks in the CV curves. This result indicates that there are two potential ranges for ORR catalytic by the LSF catalysts, the first one is from 0.8 V to 0.37 V and the second one is 0.37–0.4 V. In the first ORR range, the onset potential of LSF0.95-1000 °C is 0.69 V, which is 26 mV positive than that of LSF0.95-1100 °C (0.67 V), and 44 mV positive than that of LSF0.95-1200 °C (0.65 V), indicating a higher ORR catalytic activity of LSF0.954-1000 °C. The half wave potential values of the LSF0.95 catalysts are 0.57 V (LSF0.95-1000 °C), 0.56 V (LSF0.951100 °C) and 0.55 V (LSF0.95-1200 °C), respectively. The positive half-wave potential of LSF0.95-1000 °C further implies the better ORR activity in the first ORR range. The onset potential and half-wave potential are also compared in the second ORR range. LSF0.95-1000 °C exhibits an onset potential of 0.36 V, and a half-wave potential of 0.17 V, which are larger than 0.34 V and 0.16 V of LSF0.95-1100 °C, and 0.33 V and 0.15 V of LSF0.95-1200 °C; suggesting a better ORR activity of LSF0.95-1000 °C in the second oxygen reduction potential range. Tafel plots of the LSF0.95 catalysts in two ORR ranges are compared in Fig. 2(b) and (c). In both ORR potential ranges, the smaller Tafel slops of LSF0.95-1000 °C (74.1 mV dec^−1^, 65.5 mV dec^−1^) indicates more favorable ORR kinetics relative to those of LSF0.95-1100 °C (78.4 mV dec^−1^, 98.7 mV dec^−1^) and LSF0.95-1200 °C (87.6 mV dec^−1^, 96.6 mV dec^−1^). Fig. 2(d) exhibits the electrochemical impedance spectroscopy (EIS) of the LSF0.95 catalysts. Compared with the other LSF0.95 catalysts, the semicircle diameter of LSF0.95-1000 °C is smaller, revealing a lower electronic resistance and faster electron transfer.

**Fig. 2:**
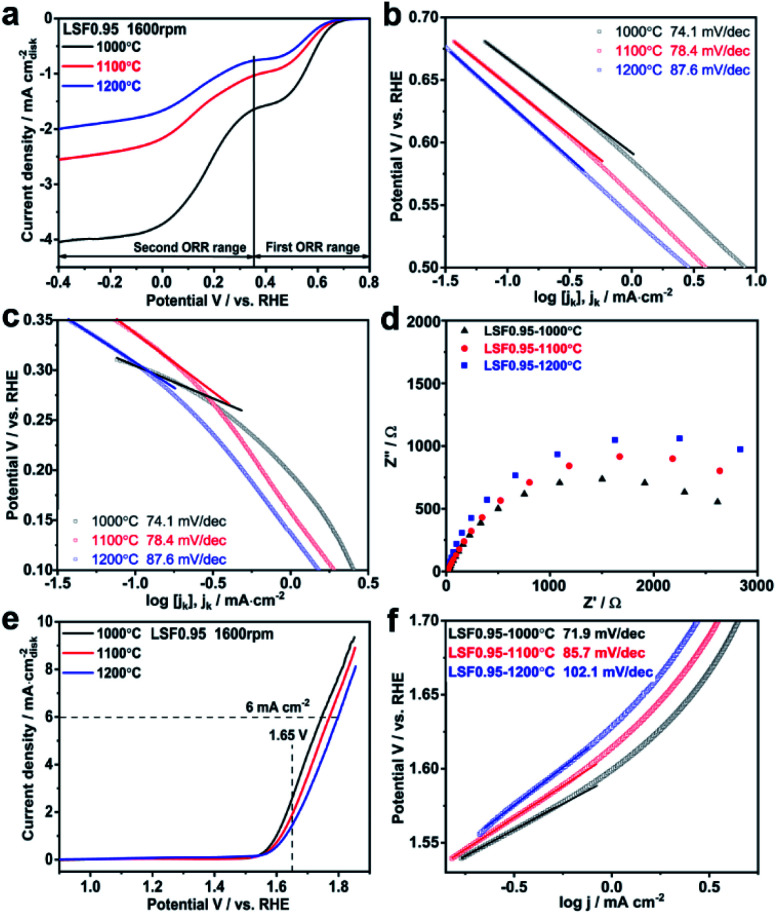
(a) LSV curves of LSF0.95 catalysts for the ORR at 1600 rpm in O_2_ saturated 0.1 M KOH with a scan rate of 10 mV s^−1^. (b) and (c) Tafel plots of LSF0.95 catalysts for two oxygen reduction ranges. (d) EIS of LSF0.95 catalysts for the ORR at 1600 rpm in O_2_ saturated 0.1 M KOH. (e) LSV curves of LSF0.95 catalysts for the OER at 1600 rpm. (f) Tafel plots of LSF0.95 catalysts for the OER.

To investigate the ORR mechanisms in these two ORR ranges, electron transfer numbers in these two ORR potential ranges are calculated from the Koutecky-Levich (K-L) plots (Fig. S5†). In the first ORR potential range, the average electron transfer number is ∼2.0, indicating an indirect 2 × 2e^−^ ORR process, and the reaction steps are shown as follow:^31^

1 O_2_ + H_2_O +2e^−^ → HO_2_^−^ + OH^−^

2 HO_2_^−^ + H_2_O + 2e^−^ → 3OH^−^

3 2HO_2_^−^ → 2OH^−^ + O_2_

The adsorbed O_2_ is reduced to the intermediate HO_2_^−^ by accepting 2e^−^ in step (1). However, the formed intermediate HO_2_^−^ is not stable, it can be further reduced to the final produce OH^−^ by accepting another 2e^−^ (2), and it also can be chemical decomposed to O_2_ again (3). The electron transfer number of LSF0.95 catalysts is ∼3.8 in the second ORR potential range, implying a direct 4e^−^ dominant ORR process. In this ORR process, the adsorbed O_2_ can be directly reduced to the OH^−^ without intermediate HO_2_^−^ generation, which is shown in eqn (4):

4 O_2_ + H_2_O + 4e^−^ → 4OH^−^

The electrocatalytic activities of LSF0.95 catalysts for OER are also investigated by RDE in N_2_-saturated 0.1 M KOH. Fig. 2(e) shows the comparison of OER LSV curves and all of them exhibit the similar features. As shown in Fig. 2(e), as the preparation temperature increases from 1000 °C to 1200 °C, the potential values at 6 mA cm^−2^ increase from 1.74 V to 1.77 V and 1.79 V; while the current densities at 1.65 V decrease gradually from 2.65 mA cm^−2^ to 1.95 mA cm^−2^ and 2.46 mA cm^−2^. These analyses indicate a higher electrocatalytic activity and a better conductivity of LSF0.95-1000 °C than the other two catalysts towards OER in 0.1 M KOH. To gain deep insight into the OER catalytic kinetics, Tafel plots are obtained from LSV curves. As shown in Fig. 2(f), the Tafel slop of LSF0.95-1000 °C is 71.9 mV dec^−1^, and it is obviously smaller than 85.7 mV dec^−1^ of LSF0.95-1100 °C and 102.1 mV dec^−1^ of LSF0.95-1200 °C, indicating a faster OER catalytic kinetics of the LSF0.95-1000 °C. EIS of the LSF0.95 catalysts are measured at 0.8 V in 0.1 M KOH and compared in Fig. S6.† The diameter of EIS semicircle increases gradually from LSF0.95-1000 °C to LSF0.95-1200 °C, which reveals a fastest charge transfer resistance of LSF0.95-1000 °C for the OER.

All the analysis above proves the best electrocatalytic activity of LSF0.95-1000 °C towards both ORR and OER in 0.1 M KOH. To investigate the influence of BET surface areas on electrocatalytic activities of LSF0.95 catalysts towards ORR and OER, LSV curves of ORR and OER per BET surface areas are compared in Fig. S7 and S8.† The LSV curves per BET surface areas exhibit the similar features with those per geometric surface areas, but the distances between LSV curves are smaller. LSF0.95-1000 °C still exhibits the best catalytic activity towards ORR and OER among three catalysts. These results imply that the BET surface areas can affect the electrocatalytic activities of the LSF0.95, but not the determined factor. The electrochemical active surface areas of LSF0.95 are obtained by measuring the electrochemical capacitance of the electrode–electrolyte interface in the non-faradaic region of cyclic voltammetry. As shown in Fig. S9,† the average value of the capacitance for LSF0.95-1000 °C, LSF0.95-1100 °C and LSF0.95-1200 °C is 0.0184 mF, 0.0132 mF and 0.0116 mF respectively. LSF0.95-1000 °C exhibits the largest reaction interface, which causes a higher ORR and OER catalytic activity. Another important reason for the outstanding catalytic activity of LSF0.95-1000 °C is the content of active site. The reaction active site in perovskite catalyst is reported to be M^3+^,^31–33^ and the higher content of M^3+^ means the better electrocatalytic activity. As discussed in XPS, LSF0.95-1000 °C possesses a larger content of Fe^3+^ than the other LSF0.95 catalysts, which can provide more reaction active sites for ORR and OER. Moreover, the content of oxygen vacancy in LSF0.95 catalysts also affects the electrocatalytic activities by influencing their conductivity and the lattice oxygen mobility.^27,34–36^ As shown in Table S4,† with the increasing of the preparation temperature, the content of the oxygen vacancy decreases, which also explains the better electrocatalytic activity of LSF0.95-1000 °C.

By comparing the LSV curves and Tafel plots of LSF0.95-1000 °C/C (20 wt% Vulcan XC-72 carbon) with commercial 20 wt% Pt/C and RuO_2_/C (20 wt% Vulcan XC-72 carbon) (Fig. S10†), the electrocatalytic activity of LSF0.95/C for the ORR is proved to be better than RuO_2_/C, but worse than 20 wt% Pt/C; while the OER activity is better than 20 wt% Pt/C, but worse than RuO_2_/C. Chronoamperometric (CA) measurements are used to investigate the electrochemical stability of LSF0.95/C for the ORR and OER, and the results are shown in Fig. S11.† After 30 hours test at 0.18 V for the ORR, the current density of LSF0.95/C keeps 99% with a negligible decrease, which is much better than that of 20 wt% Pt/C (93%). For the OER at 1.63 V, the current densities of LSF0.95/C almost keep 85% after 40 hours, and it is much larger than 64% of RuO_2_/C. These results reveal an excellent electrochemical durability of LSF0.95/C. The XRD, XPS and HRTEM results of LSF0.95 in Fig. S12 and S13† after durability measurements further identify the excellent stability. The equation Δ*E* = *E*_OER_ − *E*_ORR_ was used to calculate the overpotential between the OER at the current density of 10 mA cm^−2^ and the ORR at the current density of −1 mA cm^−2^, which is used to access the bifunctionality of the catalysts.^37,38^ The overpotential values of LSF0.95/C are compared with the reported ones in Table S5.† The Δ*E* values for LSF0.95/C, 20 wt% Pt/C and RuO_2_/C are 1.04 V, 1.10 V and 1.12 V, respectively. Obviously, the Δ*E* of LSF0.95/C is smaller than commercial noble-metal based Pt/C, RuO_2_/C and some other reported excellent perovskite-based bifunctional catalysts. The results further demonstrate the high bifunctional electrocatalytic activity of LSF0.95 in alkaline electrolyte.

The superior bifunctional ORR and OER activity and durability of LSF0.95/C motivated us to investigate its performance in realistic primary and rechargeable Zn–air batteries. Fig. 3(b) presents discharge polarization and power density curves for Zn–air batteries based on LSF0.95-1000 °C and commercial 20 wt% Pt/C catalysts. When the current density < 70 mA cm^−2^, the voltage and power density of the battery based on perovskite LSF0.95-1000 °C are very close to those of the commercial Pt/C. But when the current density > 70 mA cm^−2^, LSF0.95-1000 °C exhibits a higher voltage and power density compared with commercial Pt/C, and the peak power density could be as high as 94 mW cm^−2^ at 0.63 V, which is superior to the commercial Pt/C. The galvanostatic discharge curves shown in Fig. 3(c) clearly exhibit that the voltage of LSF0.95-1000 °C cathode (∼1.23 V) is similar to that of Pt/C cathode (∼1.24 V) in primary Zn–air battery at a current density of 10 mA cm^−2^, after 10 h discharge, no obvious voltage drop was observed until all of the Zn metal was consumed. The specific discharge capacities at different current density are shown in Fig. 3(d), at 10 mA cm^−2^, the specific capacity of LSF0.95-1000 °C based battery normalized to the mass of consumed Zn is 755 mA h g_Zn_^−1^, corresponding to a gravimetric energy density of 928 W h kg^−1^. At 30 mA cm^−2^, the specific capacity normalized to the mass of consumed Zn is ∼732 mA h g_Zn_^−1^, corresponding to a high gravimetric energy density >768 mA h g_Zn_^−1^. The rechargeable Zn–air batteries with short cycles (10 min per cycle) are performed to explore the bi-functional properties of LSF0.95-1000 °C. The batteries are running in the air at room temperature, no further oxygen or air is bubbled into the system. At 10 mA cm^−2^, the charge–discharge voltage gap for LSF0.95-1000 °C is ∼0.74 V, which is very close to voltage gap between the commercial 20 wt% Pt/C and RuO_2_/C (∼0.66 V), and it can keep this value after 10 h running (Fig. S14†). This result of rechargeable Zn–air batteries also implies a superior bifunctional electrocatalytic activity of the LSF0.95 for the ORR and OER.

**Fig. 3:**
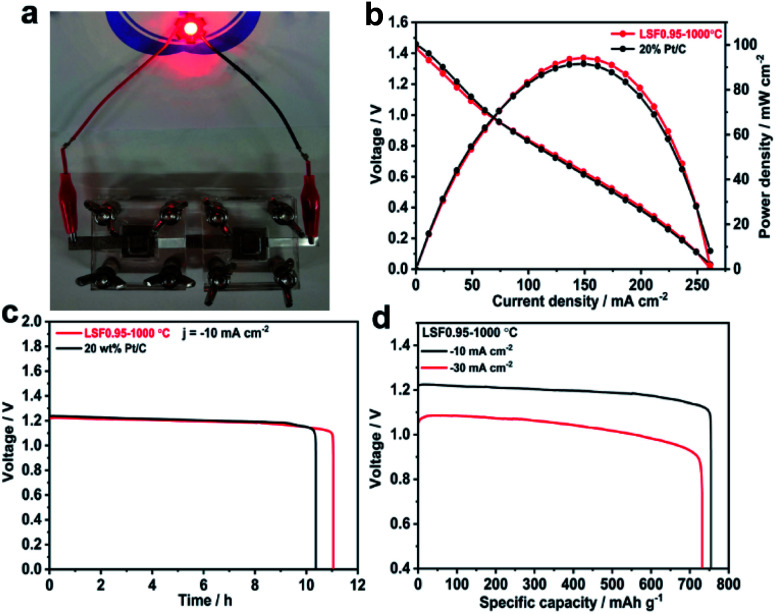
(a) Image of the assembled Zn–air battery. (b) Polarization curves and corresponding power density plot of the primary Zn–air batteries using LSF0.95-1000 °C and 20% Pt/C based cathodes in 6 M KOH. (c) The galvanostatic discharge curves of the primary Zn–air batteries using LSF0.95-1000 °C and 20% Pt/C based cathodes at the current density of 10 mA cm^−2^. (d) Specific capacities of the primary Zn–air batteries using LSF0.95-1000 °C based cathodes at the current density of 10 mA cm^−2^ and 30 mA cm^−2^.

## Conclusions

In summary, a series of (La_0.65_Sr_0.3_)_0.95_FeO_3−*δ*_ perovskite catalysts are prepared by a solid-state synthesis method at different temperatures. LSF0.95-1000 °C exhibits the highest bifunctional catalytic activity among these LSF0.95 catalysts and a smaller overpotential between ORR and OER. It also performs a stronger durability than commercial noble-metal catalysts. The LSF0.95-1000 °C catalyst is used as the bifunctional electrocatalyst in primary and rechargeable Zn–air batteries. The maximum power density of the primary Zn–air battery using LSF0.95-1000 °C can reach 94 mW cm^−2^, which is superior to the commercial Pt/C. The performance of the rechargeable Zn–air battery of (La_0.65_Sr_0.3_)_0.95_FeO_3−*δ*_-1000 °C catalyst is comparable with combined commercial catalysts Pt/C and RuO_2_. The outstanding activity can be attributed to the amount of active sites, and the higher content of oxygen vacancy.

## Conflicts of interest

There are no conflicts to declare.

## Supplementary Material

RA-011-D1RA07920D-s001
