# Supplementary material for: (La0.65Sr0.3)0.95FeO3−δ perovskite with high oxygen vacancy as efficient bifunctional electrocatalysts for Zn–air batteries
Source: RSC Adv. 2021 Dec 6;11(62):38977–81. doi: 10.1039/d1ra07920d (PMC9044435; doi:10.1039/d1ra07920d)
Supplement: RA-011-D1RA07920D-s001 [file RA-011-D1RA07920D-s001.pdf]

Electronic Supplementary Material (ESI) for RSC Advances.
This journal is © The Royal Society of Chemistry 2021

Supporting Information

$(La_{0.65}Sr_{0.3})_{0.95}FeO_{3-\delta}$ perovskite with A-site deficiency as efficient bifunctional electrocatalysts for Zn-air batteries

Liyang Luo,[a] Zhongyi Liu,[a] and Zhiyuan Wang,*[b,c]

[a]College of Chemistry, Zhengzhou University, Zhengzhou 450001, China.

[b]Henan Institute of Advanced Technology, Zhengzhou University, Zhengzhou 450001, China.

[c] Institute of Physical Chemistry, RWTH Aachen University, 52074 Aachen, Germany

Experimental Section

Chemicals

La_2O_3 (99.99%), $SrCO_3$ (≥99.9%), Fe_2O_3 (≥99%) and isopropanol (99.8%) were purchased from Sigma-Aldrich. All chemicals were used as received without further purification. Deionized (DI) water from Milli-Q System (Millipore, Billerica, MA) was used in all our experiments.

Materials preparation

$(La_{0.65}Sr_{0.3})_{0.95}FeO_{3-\delta}$ oxides were synthesized by a solid-state method. In a typical synthesis, according to the composition of $(La_{0.65}Sr_{0.3})_{0.95}FeO_{3-\delta}$, La_2O_3, $SrCO_3$, Fe_2O_3 and 100 ml isopropanol were mixed with a certain molar ratio and ball-milled for 5 h at 250 rpm. Then the isopropanol was evaporated under vacuum at 60 °C and the obtained mixture was dried at 80 °C overnight. The dried mixture was calcined in Muffle furnace at designed temperature under air for 8 h with a heating rate of 5 °C/min, after cooling down to the room temperature, the sample was grinded and stored for further use. Based on the calcined temperature, the samples were named as LSF0.95-900°C, LSF0.95-1000°C, LSF0.95-1100°C and LSF0.95-1200°C, respectively.

Materials characterization

Powder X-ray diffraction patterns were recorded on a PANalytical operating at 40 KV voltage and 15 mA current with Cu Kα radiation (λ=1.5418 Å). Rietveld refinement[19] of X-ray data was carried out by the FULLPROF code. Scanning electron microscope (SEM) was recorded on a Quanta FEG 650 at 20kV. The specific surface area was obtained by Brunauer-Emmett-Teller (BET) method from N_2 adsorption/desorption isotherms at 77 K in a relative pressure range from 0.1 to 0.3 (Quanta Chrome Instruments, USA). The particle size distributions were analyzed by laser diffraction particle size analyzer (HORIBA, LA-960). The chemical compositions were detected by inductively coupled plasma with optical emission spectroscopy (ICP-OES) (Thermo Scientific iCAP7600). X-ray photoelectron spectroscopy (XPS) was collected on ULVAC-Phi 5000 instrument equipped with an Al kα source (1.486 keV).

Electrode preparation

The thin-film working electrode was prepared by depositing 2 μl well-dispersed catalyst ink on the RDE glassy carbon (GC) electrode (3 mm, ALS Co., Ltd) and dried at room temperature. For the RRDE GC electrode (4 mm, ALS Co., Ltd), 5 μl catalyst ink was deposited. The catalyst loading for RDE and RRDE is 3.77×10^{-2} mg cm^{-2}. The catalyst ink was prepared by dispersing 10 mg catalyst and 80 μl 5 wt% Nafion (Aldrich) into 450 μl solvent (Vethanol: VDI-water = 1:1) and sonicated for 1 h. Before each use GC electrodes were polished with 1 μm polishing diamond and 0.05 μm polishing alumina (PK-3 electrode

polishing kit, ALS Co., Ltd). By comparison, the working electrodes of LSF0.95/C (consist of 80 wt% LSF0.95 and 20 wt% Vulcan XC-72), commercial Pt/C (20 wt% Pt and 80 wt% Vulcan XC-72 carbon, electrochemical purity, Sigma-Aldrich) and RuO_2/C (80 wt% RuO_2 and 20 wt% Vulcan XC-72 carbon, electrochemical purity, Sigma-Aldrich) were prepared by the same method with $(La_{0.65}Sr_{0.3})_{0.95}FeO_{3-\delta}$ catalysts. All the current density (mA cm^{-2}) showed in this paper was calculated from electrode geometric surface area without special noting.

Electrochemical measurement

Electrochemical measurements were performed with RRDE-3A (ALS Co., Ltd) and SP-300 (Bio-Logic) potentiostat by a standard three-electrode system in 0.1 M KOH solution at room temperature, using the prepared GC electrode as the working electrode, a Pt wire as the counter electrode, and a Hg/HgO (1 M NaOH) as the reference electrode. Before each test, the KOH was saturated with oxygen for at least 30 min. Cyclic voltammetry (CV) and linear sweep voltammetry (LSV) were performed at a scan rate of 10 mV s^{-1}. All current densities (mA cm^{-2}) shown in this paper are calculated from the geometric area of the electrode without special noting. The overall electron transfer number during ORR was obtained from Koutecky-Levich plots:

$$1/j = 1/j_k + 1/j_d = -1/nFkC^o - 1/0.21nFD^{2/3}v^{-1/6}C^o\omega^{1/2}$$

And, $$B = -1/0.62nFC^oD^{2/3}v^{-1/6}$$

Where j, j_k and j_d are the measured current density, the kinetic current density and the limiting current density (mA cm^{-2}), respectively; F is the Faraday constant (96485 C·mol^{-1}); D is the diffusion coefficient of oxygen in 0.1 M KOH at room temperature (1.73×10^{-5} $cm^2\cdot s^{-1}$); C^o is the saturated concentration of oxygen in 0.1M KOH (1.14×10^{-6} mol·cm^{-3}); n is the number of electrons transferred per oxygen molecule; ω is the rotating speed in rpm; v is the kinetic viscosity of the solution at room temperature (0.01 $cm^2\cdot s^{-1}$); k is the rate constant.

All kinetic current densities j_k for ORR Tafel plots were corrected by the following equation:

$$j_k = j * j_d/(j_d - j).$$

The electrochemical active surface areas (ECSA) of the LSF0.95 catalysts were estimated by measuring the electrochemical capacitance of the electrode-electrolyte interface in the non-faradaic region of cyclic voltammetry[20, 21]. The ECSA of the catalyst was calculated as:

$$ECSA = C_{dl}/C_s$$

$$i_{dl} = C_{dl} \times v$$

Where C_{dl} is the specific capacitance of the electrode double layer (mF); v ($V\ s^{-1}$) is the scan rate; Cs is the specific capacitance in 0.1 M KOH[21].

All potentials in this paper were converted to the reversible hydrogen electrode (RHE) *via* Nernst equation:

$$E_{RHE} = E_{Hg/HgO} + 0.059\ V \times pH + E^{\circ}_{Hg/HgO}$$

Where $E_{Hg/HgO}$ is the measured potential and the $E^{\circ}_{Hg/HgO}$ is the standard potential of Hg/HgO at 25 °C (0.098 V).

Zn-air battery performance

Primary Zn-air batteries were tested in home-built electrochemical cells, where 6.0 M KOH was used as the electrolyte, and a zinc plate (thickness = 0.25 mm) as the anode. The cathode was prepared by uniformly coating the prepared catalytic ink onto carbon paper; the mass loading was 2 mg cm^{-2}, followed by drying at 60 °C overnight.

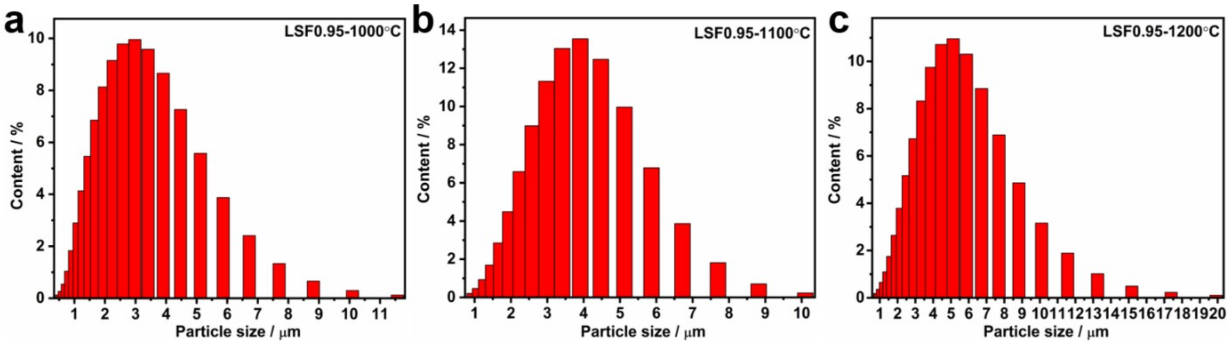

Figure S1. Histograms of particle size distribution for LSF0.95 calcined at 1000 °C (a), 1100 °C (b)and 1200 °C (c).

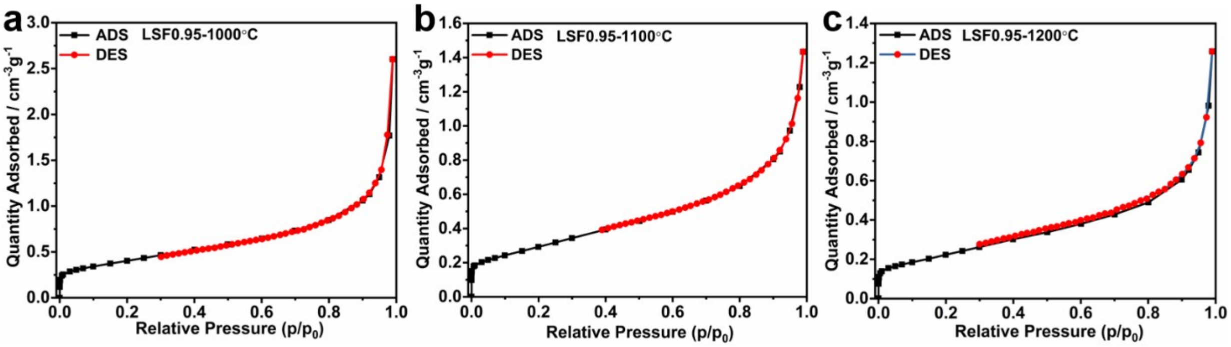

Figure S2. Nitrogen adsorption-desorption isotherms of the LSF0.95 catalysts calcined at 1000 °C (a), 1100 °C (b)and 1200 °C (c).

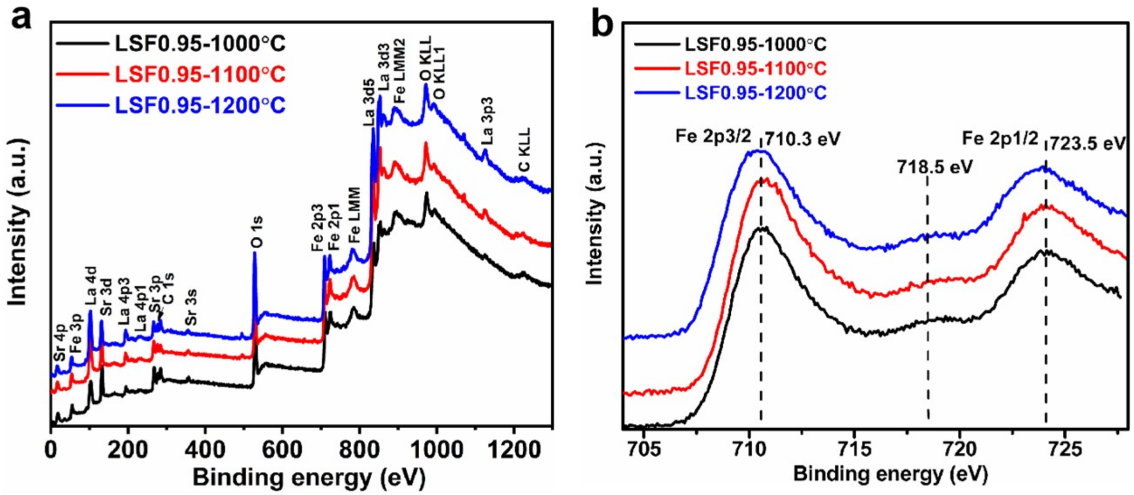

Figure S3. (a) XPS survey scans of the LSF0.95 catalysts. (b) High resolution XPS spectra of Fe for LSF0.95 catalysts.

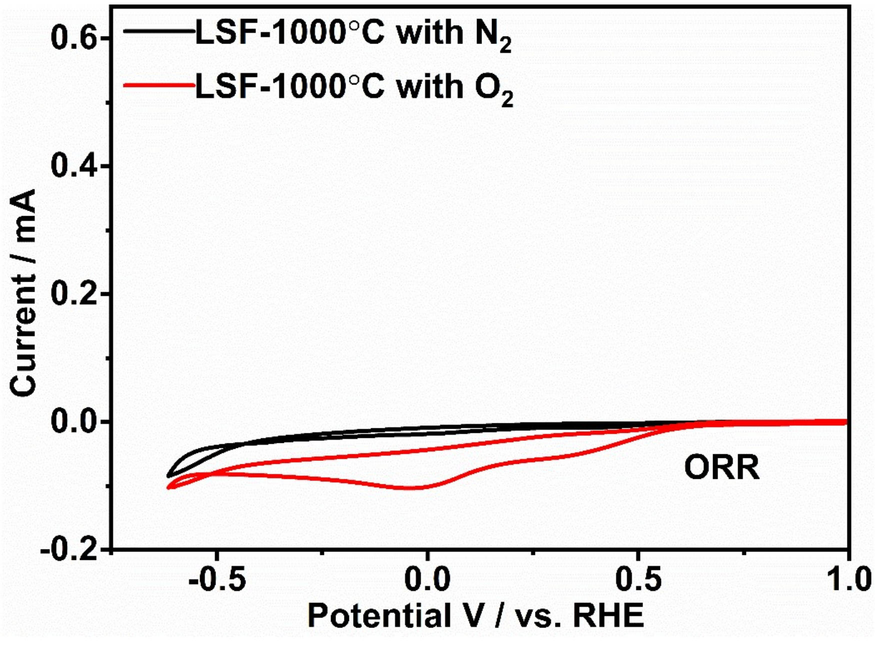

Figure S4. CV curve of the LSF0.95 catalyst on RDE in N_2 and O_2 saturated 0.1 M KOH at a scan rate of 10 mV/s.

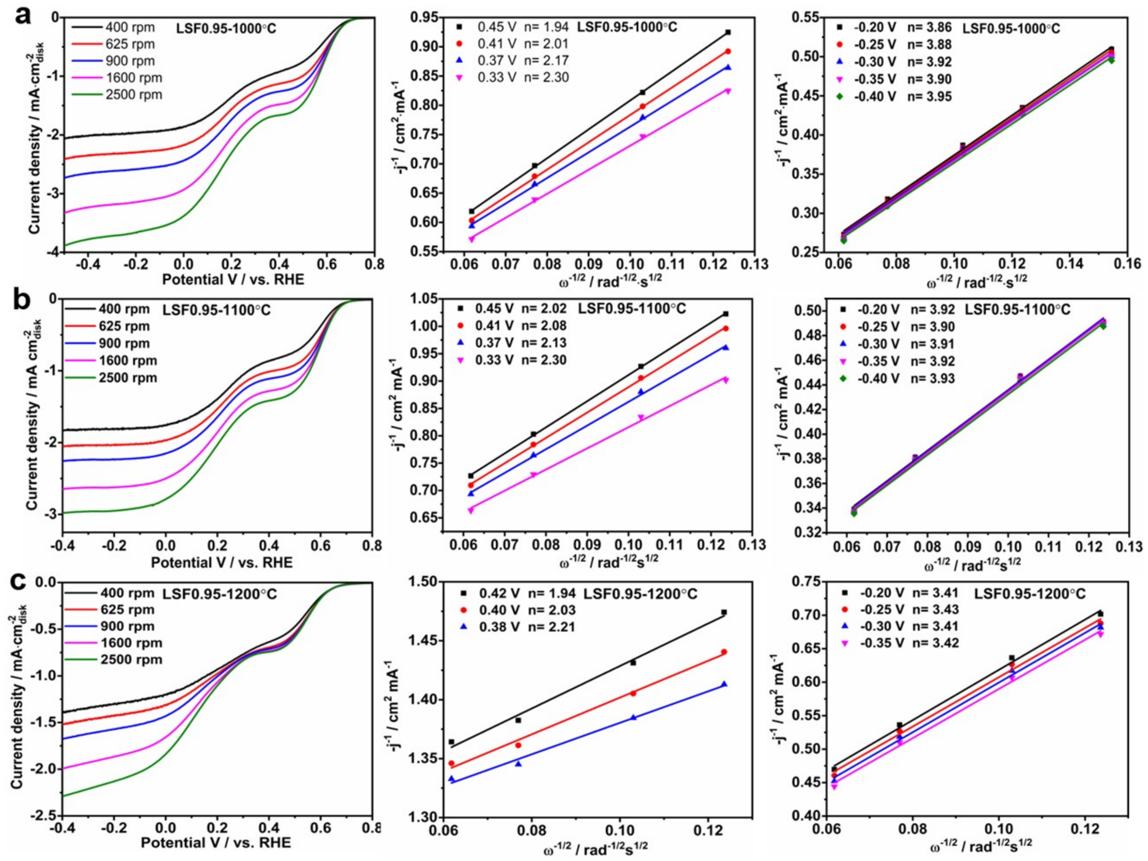

Figure S5. LSV curves on RDE at different rotation speed in O_2 saturated 0.1 M KOH at a scan rate of 10 $mV{\cdot}s^{-1}$ and K-L plots of two oxygen reduction ranges for: LSF0.95-1000°C (a), LSF0.95-1100°C (b), LSF0.95-1200°C (c).

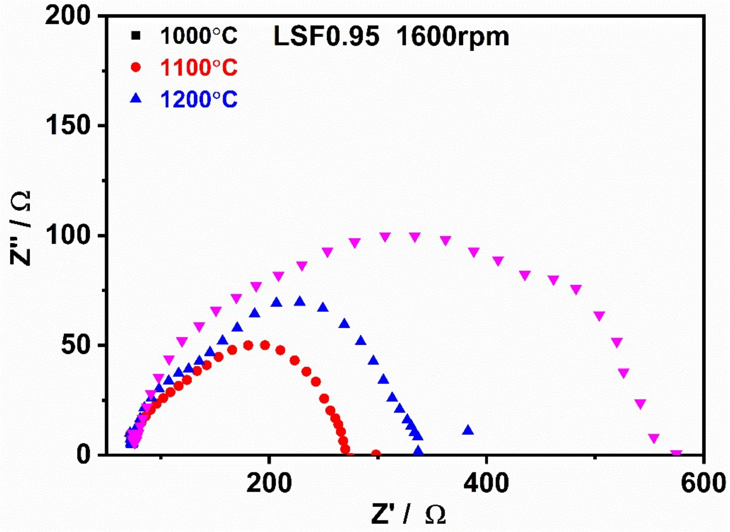

Figure S6. EIS of LSF0.95 catalysts recorded at 1.68 V versus RHE under the influence of an AC voltage of 10 mV.

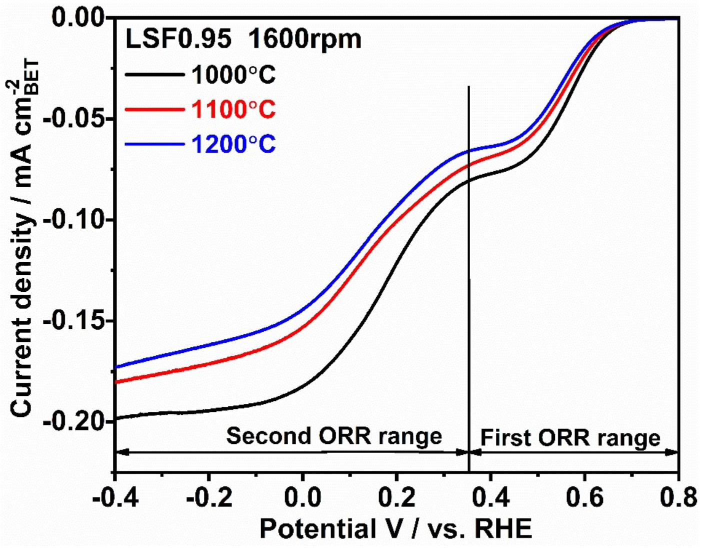

Figure S7. ORR specific activities of LSF0.95 catalysts per BET surface area.

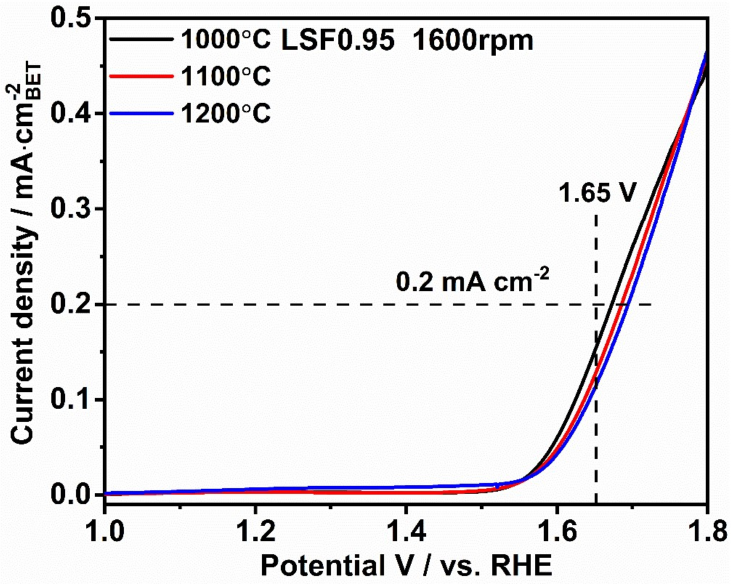

Figure S8. OER specific activities of the LSF0.95 catalysts per BET surface area.

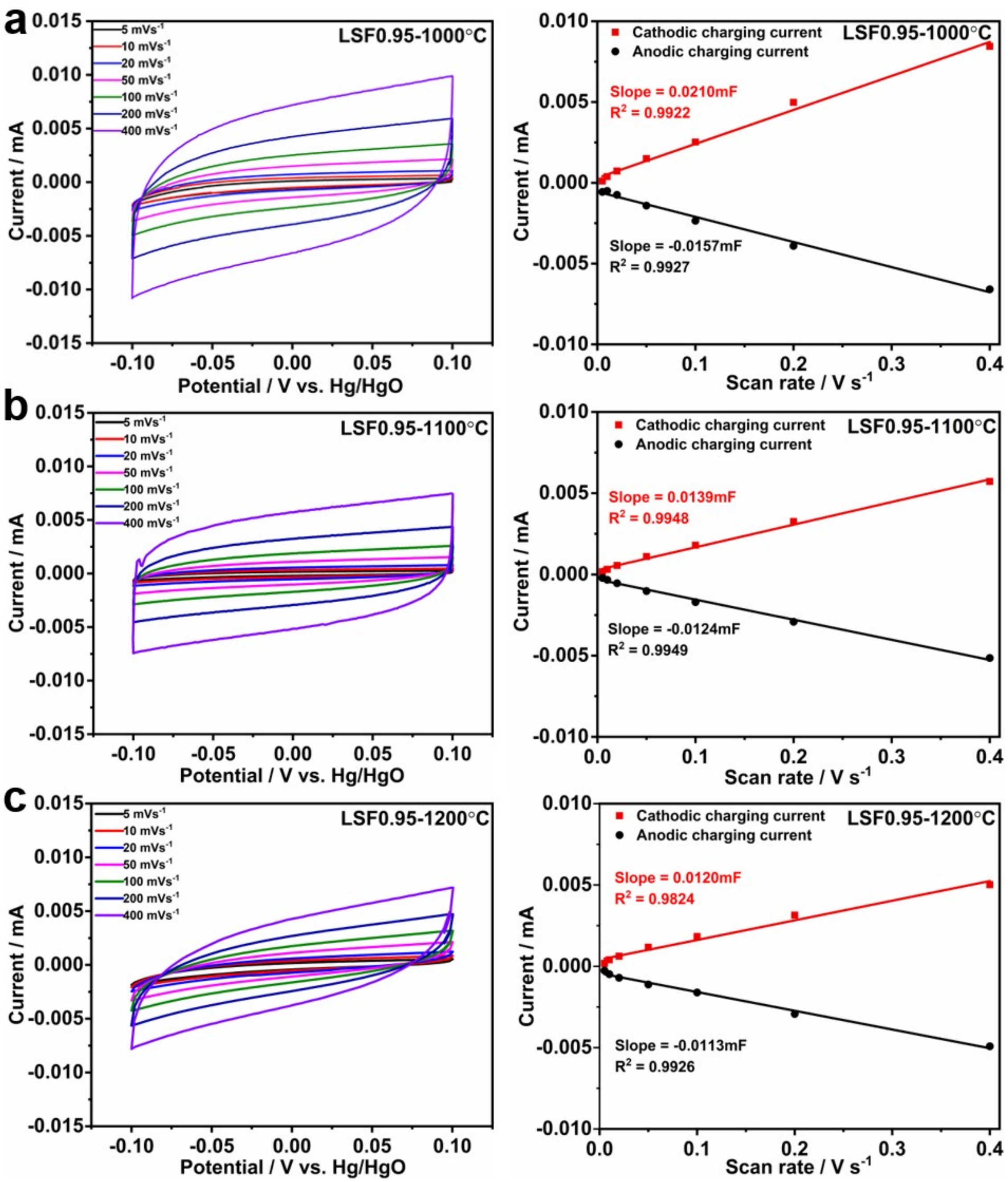

Figure S9. Double-layer capacitance measurements for determining electrochemical active surface area for LSF0.95 catalysts from CV in N_2 saturated 0.1 M KOH. The double layer capacitance is taken as the average of the absolute value of the slope of the linear fits to the data.

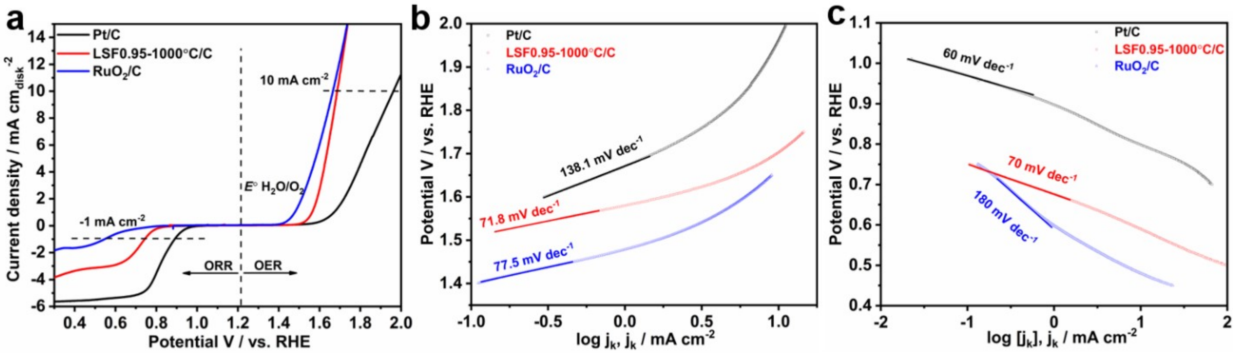

Figure S10. (a) The comparison of ORR and OER LSV curves for the Pt/C, LSF0.95/C (1000 °C) and RuO_2/C in O_2 saturated 0.1 M KOH. Tafel plots of Pt/C, LSF0.95/C (1000 °C) and RuO_2/C obtained from ORR (b) and OER (c) LSV curves.

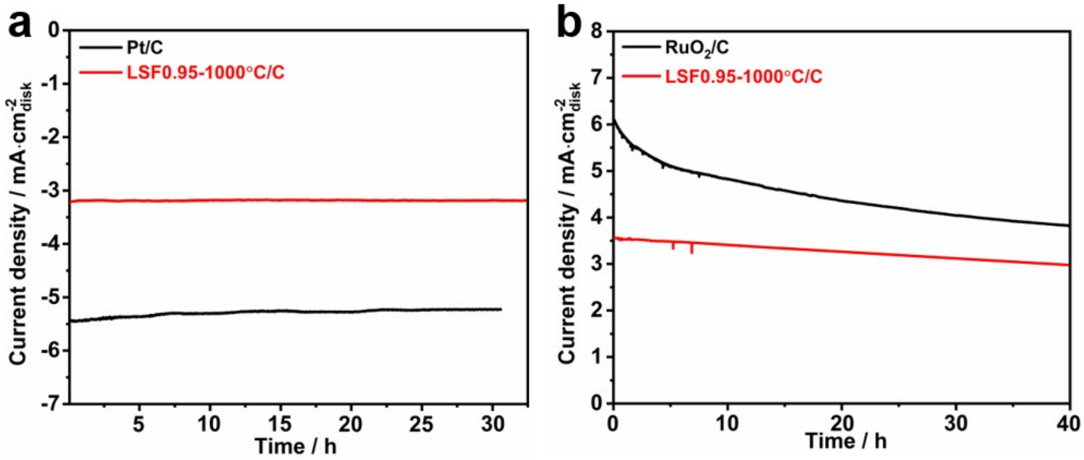

Figure S11. (a) The Chronoamperometric (CA) measurements of Pt/C and LSF0.95/C (1000 °C) in O_2 saturated 0.1 M KOH at 1600 rpm and 0.18 V. (b) The Chronoamperometric (CA) measurements of RuO_2/C and LSF0.95/C (1000 °C) in O_2 saturated 0.1 M KOH at 1600 rpm and 1.63 V.

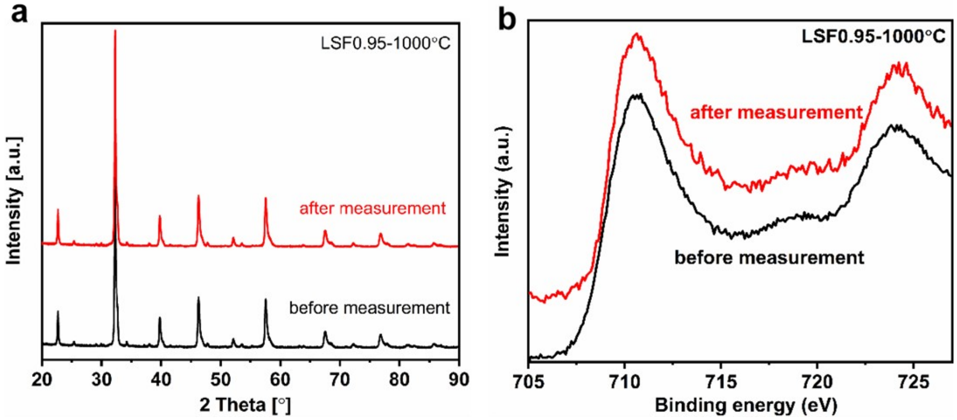

Figure S12. (a) The XRD patterns and (b) XPS spectra of Fe 2p species of LSF0.95-1000°C before and after electrochemical stability measurement.

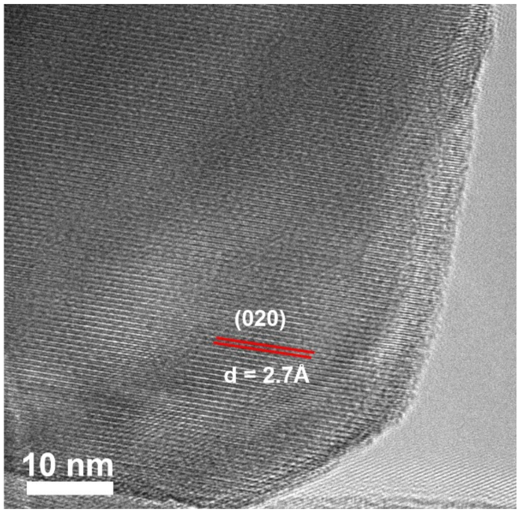

Figure S13. The HRTEM image of LSF0.95-1000°C after electrochemical stability measurement.

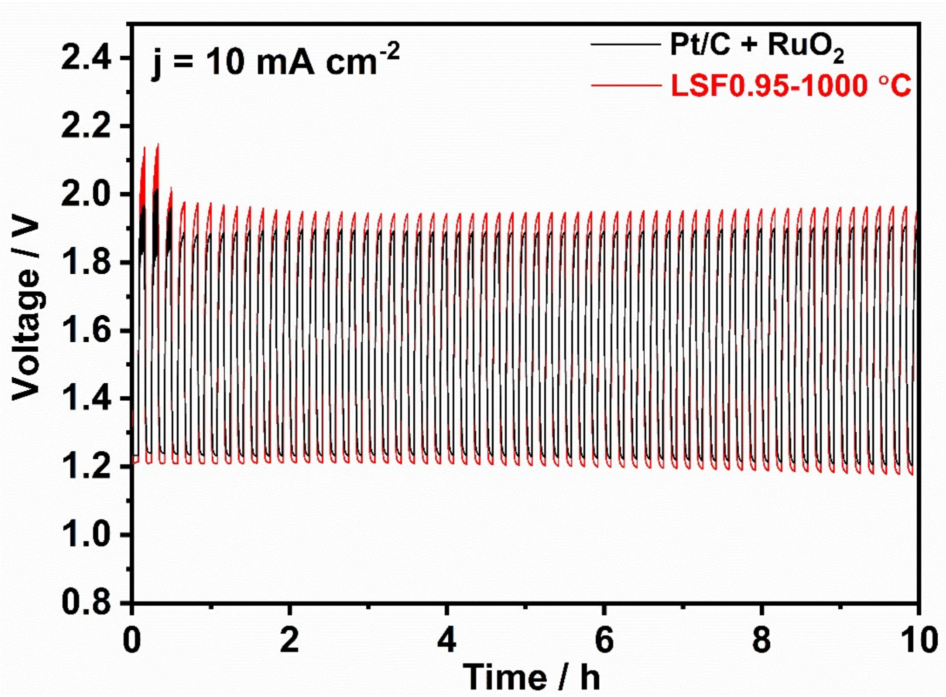

Figure S14. Cycling performance of the rechargeable Zn-air battery using LSF0.95-1000°C (red) compared with the one using Pt/C and RuO2 (black).

Table S1. Chemical composition s of the $(La_{0.65}Sr_{0.3})_{0.95}FeO_{3-\delta}$ catalysts prepared at 1000 °C, 1100 °C and 1200 °C.

Nominal composition	The concentration of metal ions ($mg\ ml^{-1}$)						ICP-OES composition
	La	SD	Sr	SD	Fe	SD	
LSF0.95-1000 °C	40.36	0.16	11.43	0.04	26.19	0.07	$La_{0.6195}Sr_{0.2782}FeO_{3-\delta}$
LSF0.95-1100 °C	40.33	0.16	11.41	0.05	26.16	0.07	$La_{0.6198}Sr_{0.2780}FeO_{3-\delta}$
LSF0.95-1200 °C	40.31	0.19	11.39	0.06	26.18	0.15	$La_{0.6190}Sr_{0.2773}FeO_{3-\delta}$

Standard deviation is based on standard deviation of triplicate measurements.

Table S2. The specific surface areas of LSF0.95 perovskite catalysts.

Sample	S_{BET} ($m^2\ g^{-1}$)
LSF0.95-1000°C	1.241
LSF0.945-1100°C	1.070
LSF0.945-1200°C	0.916

S_{BET} : Specific surface area calculated by Brunauere-Emmette-Teller model.

Table S3. Iron valences, oxygen species of the different LSF0.95 catalysts analyzed by XPS.

Sample	Fe^{3+} (%)	Fe^{4+} (%)	Lattice O^{2-} (%)	Lattice O_2^{2-}/O^- (%)	$-OH/O_2$ (%)	H_2O (%)
1000°C	59.91	40.09	47.35	26.53	21.68	4.45
1100°C	50.92	49.08	48.37	29.80	17.63	4.20
1200°C	41.22	58.78	40.74	32.83	22.58	3.85

Table S4. The content of oxygen vacancy and calculated formula based on XPS results.

Sample	Fe^{3+} (%)	Fe^{4+} (%)	Oxygen vacancy / δ	Calculated formula
1000°C	59.91	40.09	0.0916	$(La_{0.65}Sr_{0.3})_{0.95}FeO_{2.9084}$
1100°C	50.92	49.08	0.0467	$(La_{0.65}Sr_{0.3})_{0.95}FeO_{2.9533}$
1200°C	41.22	58.78	-0.0018	$(La_{0.65}Sr_{0.3})_{0.95}FeO_{3.0018}$

Table S5. Comparison of the bifunctional catalytic activity for LSF0.95 catalyst, precious-metal based and some other excellent perovskite bifunctional catalysts reported in the literatures. All the catalysts in the table were tested in oxygen saturated 0.1 M KOH solution.

Catalysts	E_{ORR} (V) at -1 mA cm^{-2}	E_{OER} (V) at 10 mA cm^{-2}	ΔE (V, E_{OER}-E_{ORR})	Ref
LSF0.95/C	0.66 vs. RHE	1.70 vs. RHE	1.04	this work
Pt/C	0.89 vs. RHE	1.99 vs. RHE	1.10	this work
RuO_2	0.55 vs. RHE	1.67 vs. RHE	1.12	this work
IrO_2	0.38 vs. RHE	1.70 vs. RHE	1.32	S1
$L0.95FeO_3$	0.58 vs. RHE	1.64 vs. RHE	1.06	S2
$La_{0.6}Sr_{0.4}CoO_{3-\delta}$-ball milled	-0.19 vs. Hg/HgO	0.89 vs. Hg/HgO	1.08	S3
$CaMnO_{2.77}$ nanoparticle	0.86 vs. RHE	>1.95 vs. RHE	>1.09	S4
$BaTiO_{3-x}$	0.72 vs. RHE	>1.90 vs. RHE	>1.18	S5
$La_{0.58}Sr_{0.4}Co_{0.2}Fe_{0.8}O_{3-\delta}$/ nitrogen-doped graphene	0.67 vs. RHE	1.72 vs. RHE	1.05	S6
$LaNi_{0.85}Mg_{0.15}O_3$	-0.31 vs. SCE	0.84 vs. SCE	1.15	S7
$LaTi_{0.65}Fe_{0.35}O_{3-\delta}$/ nitrogendoped carbon nanorods	0.78 vs. RHE	1.81 vs. RHE	1.03	S8

References:

S1. Rincón, R. A.; Masa, J.; Mehrpour, S.; Tietz, F.; Schuhmann, W. Activation of oxygen evolving perovskites for oxygen reduction by functionalization with Fe-N x/C groups. Chem. Commun 2014, 50, 14760-14762.

S2. Zhu, y. l.; Zhou, W.; Yu, J.; Chen, Y. B.; Liu, M. L.; Shao, Z. P., Enhancing Electrocatalytic Activity of Perovskite Oxides by Tuning Cation Deficiency for Oxygen Reduction and Evolution Reactions. Chem. Mater. 2016, 28, 1691-1697.

S3. Oh, M. Y.; Jeon, J. S.; Lee, J. J.; Kim, P.; Nahm, K. S.; The bifunctional electrocatalytic activity of perovskite $La_{0.6}Sr_{0.4}CoO_{3-\delta}$ for oxygen reduction and evolution reactions. RSC Adv. 2015, 5, 19190-19198.

S4. Du, J. Zhang, T. R.; Cheng, F. Y.; Chu, W. S.; Wu, Z. Y.; Chen, J.; Nonstoichiometric perovskite $CaMnO_{3-\delta}$ for oxygen electrocatalysis with high activity. Inorg. Chem. 2014, 53, 9106-9114.

S5. Chen, C.-F.; King, G.; Dickerson, R. M.; Papin, P. A.; Gupta, S.; Kellogg, W. R.; Wu, G. Oxygen-deficient BaTiO3-x perovskite as an efficient bifunctional oxygen electrocatalyst. Nano Energy 2015, 13, 423-432.

S6. Park, H. W.; Lee, D. U.; Zamani, P.; Seo, M. H.; Nazar, L. F.; Chen, Z. W. Electrospun porous nanorod perovskite oxide/nitrogen-doped graphene composite as a bi-functional catalyst for metal air batteries. Nano Energy 2014, 10, 192-200.

S7. Du, Z. Z.; Yang, P.; Wang, L. ; Lu, Y. H.; Goodenough, J. B.; Zhang, J.; Zhang, D. W. Electrocatalytic performances of $LaNi_{1-x}Mg_xO_3$ perovskite oxides as bi-functional catalysts for lithium air batteries. J. Power Sources 2014, 265, 91-96.

S8. Prabu, M.; Ramakrishnan, P.; Ganesan, P.; Manthiram A.; Shanmugam, S. $LaTi_{0.65}Fe_{0.35}O_{3-\delta}$ nanoparticle-decorated nitrogen-doped carbon nanorods as an advanced hierarchical air electrode for rechargeable metal-air batteries. Nano Energy 2015, 15, 92-103.
